# ProtNet: a tool for stochastic simulations of protein interaction networks dynamics

**DOI:** 10.1186/1471-2105-8-S1-S4

**Published:** 2007-03-08

**Authors:** Massimo Bernaschi, Filippo Castiglione, Alessandra Ferranti, Caius Gavrila, Michele Tinti, Gianni Cesareni

**Affiliations:** 1 Istituto per le Applicazioni del Calcolo "M. Picone", CNR, V.le del Policlinico 137, Rome, Italy; 2 Department of Biology, University of Rome Tor Vergata, Via della Ricerca Scientifica, Rome, Italy

## Abstract

**Background:**

Protein interactions support cell organization and mediate its response to any specific stimulus. Recent technological advances have produced large data-sets that aim at describing the cell interactome. These data are usually presented as graphs where proteins (nodes) are linked by edges to their experimentally determined partners. This representation reveals that protein-protein interaction (PPI) networks, like other kinds of complex networks, are not randomly organized and display properties that are typical of "hierarchical" networks, combining modularity and local clustering to scale free topology. However informative, this representation is static and provides no clue about the dynamic nature of protein interactions inside the cell.

**Results:**

To fill this methodological gap, we designed and implemented a computer model that captures the discrete and stochastic nature of protein interactions. In ProtNet, our simplified model, the intracellular space is mapped onto either a two-dimensional or a three-dimensional lattice with each lattice site having a linear size (5 nm) comparable to the diameter of an average globular protein. The protein filled lattice has an occupancy (*e.g*. 20%) compatible with the estimated crowding of proteins in the cell cytoplasm. Proteins or protein complexes are free to translate and rotate on the lattice that represents a sort of naïve unstructured cell (devoid of compartments). At each time step, molecular entities (proteins or complexes) that happen to be in neighboring cells may interact and form larger complexes or dissociate depending on the interaction rules defined in an experimental protein interaction network. This whole procedure can be seen as a sort of "discrete molecular dynamics" applied to interacting proteins in a cell.

We have tested our model by performing different simulations using as interaction rules those derived from an experimental interactome of *Saccharomyces cerevisiae* (1378 nodes, 2491 edges) and we have compared the dynamics of complex formation in a two and a three dimensional lattice model.

**Conclusion:**

ProtNet is a cellular automaton model, where each protein molecule or complex is explicitly represented and where simple interaction rules are applied to populations of discrete particles. This tool can be used to simulate the dynamics of protein interactions in the cell.

## Background

Cell structure and physiology is governed by the precise wiring of the protein interaction network in the cell. In principle, if we had quantitative information about the kinetic constants describing the association and dissociation of all pairs of proteins in a proteome, we could attempt to combine it with information about interactions with small molecules, membranes and nucleic acids to model an electronic cell. This in turn could be used to ask very specific questions in order to test our understanding of the mechanisms underlying cell organization and its dynamic response to external stimuli.

This strategy is computationally very demanding and, in its general formulation, hindered by the limitations of current technologies, at least in a foreseeable future. Currently, the main limitation is the lack of appropriate experimental technologies to produce accurate data at a global genomic level. If precise and complete data are required for carrying out simulations that have biological relevance, then it is unlikely that we will achieve useful cell models in the near future. It is possible, however, that even partial, noisy and/or qualitative data would be sufficient to develop models that can answer low-resolution questions.

Recent technological advances have offered the opportunity to accumulate extensive information about the protein interaction networks in several organisms. In particular the yeast interactome has been looked at from several perspectives and using complementary approaches [1-4]. Although these data sets are noisy and include a sizeable fraction of false positives and false negatives, we have, at this time, the best picture ever of the possible interactions occurring in a living organism.

These types of data are usually represented as a graph where nodes (proteins) are connected by edges if the proteins in question have been shown to interact. Similarly to many social and technological networks, protein-protein interaction (PPI) networks, are not random in many aspects, displaying properties that are characteristic of "hierarchical" networks combining modularity and local clustering to scale free topology [5].

The graph representation is informative since it allows us to visualize all the possible connections between the proteins in a cell. However, it is not possible to extract some biologically relevant information such as the complexes that are preferentially formed in the cell under different conditions or their compositional distribution. Finally, the graph representation does not have spatial and temporal resolution since it delineates an average over several conditions and time steps. In other words graphs render the static dimension of the protein interaction network and do not capture the dynamic nature and the non-homogeneous spatial distribution of the interactions occurring in a cell.

We present here the initial characterization of a cellular automata model that uses as input experimentally derived protein interaction graphs and, by treating explicitly each protein or complex, produces a full dynamic picture of protein complex assembly and disassembly within a stylized cell.

Although some recent reports, appearing in the literature, attempted to describe the dynamics of protein interactions [6-9], none of these represents a full dynamic model permitting the simulation of the complete set of interactions occurring *in vivo *in the four spatio-temporal dimensions.

## Results

ProtNet is a dynamic stochastic model developed to simulate protein interactions with full spatial and temporal resolution. Proteins diffuse freely in a lattice space and, whenever they reside in neighboring sites, they may form a complex depending on the interaction rules contained in an input interaction graph. The simulations presented in this report are based on the protein interaction network of the yeast *Saccharomyces cerevisiae*. Protein interaction databases store more than 40,000 interactions between yeast proteins [10]. Most of them, however, are derived from high throughput experiments, producing an inherent high level of false interaction information [11]. To minimize false positives, we utilized a high-quality yeast interaction network derived by considering only interactions described by more than one method[12] (Additional File 1). The resulting interactome data set contains 2,491 high-confidence interactions between 1378 proteins. This corresponds to an average of 3.6 interactions per protein.

In this first implementation of the model we make the following simplifications (see also methods for more details):

• The simulation space is a *domain *of the cell that does not contain compartments and is not limited by a membrane.

• All the proteins are seeded onto the lattice at the same concentration.

• Protein concentrations are constant. We do not model protein synthesis and protein degradation.

• Interacting proteins have the same probability of forming a complex, and each bond in a complex can break at each time step with the same rate constant.

• Proteins diffuse at the same rate irrespective of size.

All these simplifications are not implicit in the formulation of the model and could be removed upon the availability of new experimental data, allowing new biological questions to be asked. Although this may seem major simplifications, the basic version of the model presented here is appropriate to ask simple questions about cell organization and protein association dynamics.

### Comparison of association kinetics and complex composition at equilibrium in the two and three-dimensional models

We first compared a 2D and a 3D model. The 2D cell model is represented by a square (L = 352) containing 123,904 sites of hexagonal geometry. Considering the linear dimensions of the lattice site as 5 nm, the diameter of an average globular protein, the size of our bi-dimensional simulation space is approximately 1.8 μm, the approximate size of a two-dimensional slice of a bacterium. The 3D model is a cube containing 125000 cubic sites (50 sites for each side). Thus both simulations use a comparable number of lattice sites. If the size of each small cubic site is 5 nm, our 3D model has linear dimensions of 0.25 μm.

Both lattices were seeded with 2 × 10^4 ^monomers at random positions. This corresponds to a lattice occupancy of about 20% and a protein concentration of 5 mM, which is comparable with the estimated crowding of a eukaryotic cell[13]. Since the network contains 1378 unique protein species, each protein is, on average, represented 18 times and its final concentration is approximately 3.6 μM. In preliminary experiments we adjusted the probability of associations and dissociations to p_on _= 0.7 and p_off _= 0.002 respectively in order to have approximately one half of the proteins involved in the formation of complexes at equilibrium.

**Figure 1:**
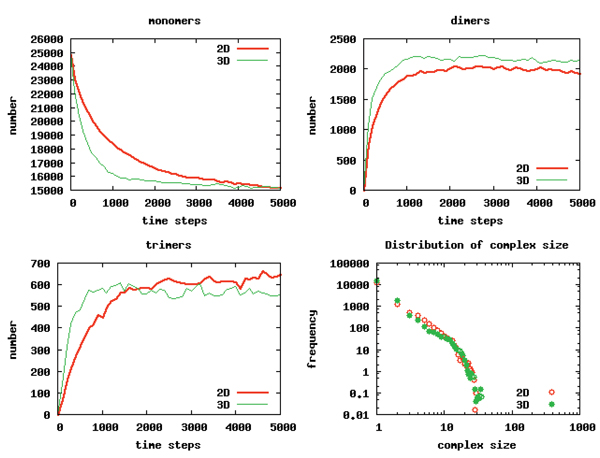
**Kinetic of complex formation in the 2D and 3D models**. The parameters of the simulation were as described in the main text. Although the simulations were carried out for 100000 steps, only the initial 5,000 steps are reported in this figure. Panel A, B and C represent the kinetics of monomer disappearance and dimer or trimer formation respectively. Panel D shows the complex size distribution at equilibrium in a double logarithmic scale. The number of complexes of any given size was averaged over last 1000 time steps; as a consequence large complexes may have, on average, an abundance of less than one.

Although the simulations were carried out for 10,000time steps, the concentration of monomers, dimers and trimers already plateau after 5000 steps (Figure 1).

Perhaps not surprisingly, the monomers were three times as fast to find their partners in the 3D than in the 2D model. Also dimers and trimers were accumulated with a faster kinetic in the 3D model. However, after 100,000 steps, the complex size distribution at equilibrium is not substantially different. The minor but significant differences observed in the number of complexes of size ~5 is difficult to rationalize. This seems to indicate that multi-protein complexes of medium size are more likely to be formed in two-dimension while dimers, trimers and very large complexes are equally probable in 2 and 3D. The experiments described in the following sessions were all carried out using a 3D lattice corresponding to a cube of linear dimensions of 0.25 μm containing 1.25 x 10^5 ^lattice sites.

### Association kinetics and complex size distribution as a function of the probability of association and dissociation

**Figure 2:**
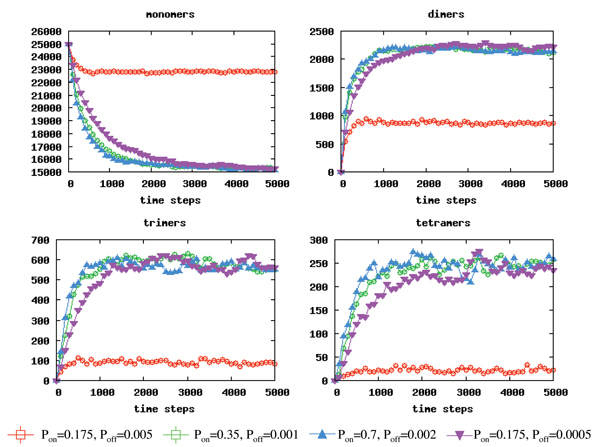
**Association kinetics for different probability of association and dissociation **. The 3D model was used for four different simulations in which the probabilities of association and dissociation were varied as indicated underneath the graphs. In three simulations (violet and cyan triangles and green circles) the ratio between P_on _and P_off _was kept constant whereas, in the run reported with the red curve, this ratio was varied to observe its impact on the concentration of the different complexes at equilibrium. The lattice sites were seeded with proteins with an occupancy of 20%.

The kinetic of complex formation depends on the two parameters P_on _defining the probability that two proteins form a complex, upon encounter, and P_off _describing the probability that any bond between two proteins forming a complex break at each time step. As shown in Figure 2 the initial rate of complex formation depends linearly on P_on _while the concentration of free monomers and complexes at equilibrium only depends on the ratio P_on_/P_off_. In the following simulations we will use the values of 0.7 and 0.002 for P_on _and P_off _respectively. In the conditions of our model these figures correspond to a dissociation constant (K_D_) of the order of micromolar.

### Association kinetics at varying molecular crowding

Protein density is another factor likely to influence the kinetics of complex formation. Higher density should increase the likelihood of protein encounter and, as a consequence, should favor complex formation. On the other hand molecular crowding should slow down diffusion and, in the long range, when large complexes are formed, prevent exploration of the cellular space. In order to evaluate the effect of molecular crowding, we performed three simulations differing in the number of monomers initially seeded in the lattice.

**Figure 3:**
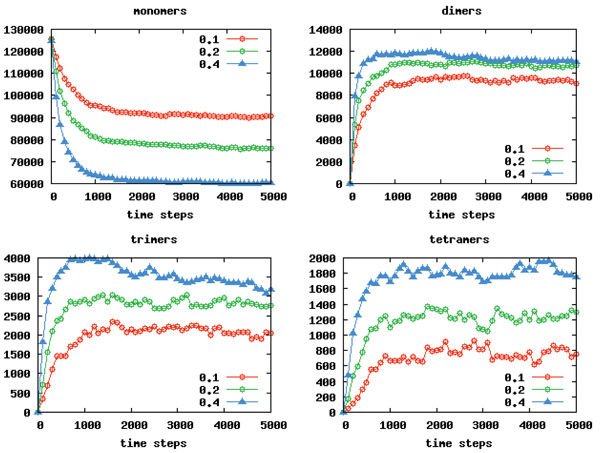
**Association kinetics for different occupancy values**. The 3D model was used for three different simulations in which the number of proteins initially seeded were varied to reach a final occupancy of 10%, 20% and 40%. Every 100 time steps the number of monomers, dimers, trimers and tetramers were counted and plotted as a function of the time steps. The figures in the ordinates represent the number of molecules at occupancy 10%. The curves for occupancies of 0.2 and 0.4 were adjusted accordingly for sake of clarity and comparison to take into account the different number of starting monomers in the grid.

For these studies, we focused on the 3D model. Figure 3 shows the kinetics of monomers, dimers, trimers and tetramers when 10, 20 and 40% of the total lattice sites are occupied. As expected, at least in the initial time steps, high protein concentration increases the probability of each protein running into one of its partners and, as a consequence, increases the probability of forming a complex. We have not observed any negative effect of molecular crowding on complex formation under these conditions, although we anticipate that the effective molecular diffusion in the cell cytoplasm should decrease at increasing protein density.

### Input and output graph

In the input graph the different proteins are joined by a varying number of edges. We were interested in investigating how node (protein) degree and other "static" graph properties are reflected in the actual number of links formed among the different members of any protein species in the cell model. To this end we examined all the interactions at quasi equilibrium after a large number of simulation steps. We then constructed a quantitative graph where the edges between any two proteins are weighted by the fraction of times the two partners take part in the specific interaction. As an example, if protein A is linked to protein B and C in the input graph we count the number of times we find a protein member A associated to B and C respectively and in the resulting graph we associate the two edges (A-B and A-C) with a figure proportional to the fraction of A proteins participating in the two interactions.

**Figure 4:**
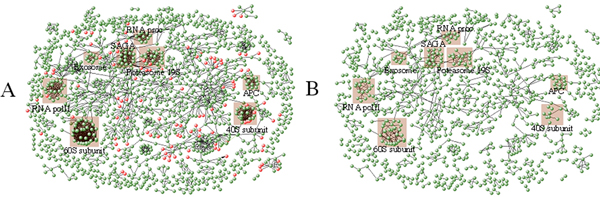
**Input and output graphs**. The graph in panel A is based on the high confidence interactome that was used as input in our simulations. Pink spheres represent proteins that, at the end of 50000 steps, were not found to be involved in the formation of any complex in a specific simulation run. Large highly connected protein "clusters" that can be mapped to physiologically relevant complexes are outlined with a brown background and identified. In panel B we have filtered the input graph (panel A) by eliminating the edges representing interactions in which the partner proteins are found less than 10% of the times. The graphs were displayed with the Visant visualization software [21].

The graph in Figure 4A represents the protein interaction network provided as input to our cell model. After 50,000 steps not all the interactions are found equally frequent in the cell model. For instance the proteins drawn in red (panel A) were never found associated to any other protein. Although the identity of these proteins may vary in different repeats of a simulation seeded with the same number of proteins, the "non-interacting" proteins, on average, share the property of being linked to a single protein connected to several partners (hubs).

Furthermore, the graph in Figure 4A is quantitative and can be filtered based on the weight of each edge. In panel B for instance, we only show the edges with a weight higher than 0.1: at least 10% of the partner proteins participate in the formation of that specific binary complex. Although, as outlined below, a detailed discussion of the biological implications of this output graph would require a more reliable and quantitative input "interactome", already this simple example sketches the formation of well characterized biological complexes and delineate the interactions that are more likely to form within the complex (Figure 4A and 4B).

**Figure 5:**
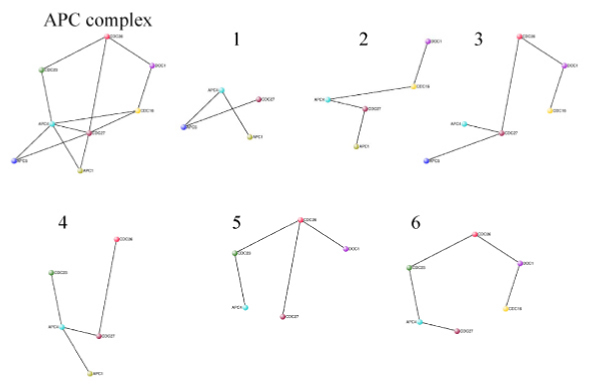
**Different topologies of APC related sub-complexes**. The graph at the top left of the figure represents the interactions between the proteins forming the Anaphase Promoting Complex (APC). The remaining graphs depict the largest complexes containing the protein CDC27 that were observed at a random specific step toward the end of the simulation (100000 steps).

Next we asked about the spread in composition and topology within the complexes that dynamically form and break during the simulation. To this end we focused on the protein CDC27 a subunit of the Anaphase Promoting Complex [14] and we listed the APC sub-complexes containing the CDC27 protein. Some examples are illustrated in Figure 5. We conclude, that in our model, the entire APC complex is never assembled in its entirety and that many possible sub-complexes form without any apparent preference for a "core complex". Although a difference in complex composition could be an inherent characteristic of the "physiological" protein interaction mesh inside the cell, these results could indicate certain limitations of both the available input data and our model.

## Discussion

The electronic cell is considered the "grail" of the field of Systems Biology, The success of this quest could reveal new perspectives for using computer simulations to accelerate biological research. Several attempts have been reported to develop simulation tools that can account not only for the stochastic nature of molecular interactions, but also for the spatial heterogeneity of cellular compartments (for reviews see [15,16]).

However, set aside the computational hurdles, these types of approaches are limited by our relative ignorance of the parameters affecting the functional interaction between proteins. A realistic simulation of cell physiology would require (at least) genome wide information about protein concentrations combined with integration of a quantitative protein interaction network with the metabolic and gene regulatory networks. These data are currently not available and are not likely to be available in a foreseeable future. Nevertheless, simple computational models may help to reveal general organizing principles from the available noisy and qualitative information.

We aim at investigating, by means of a combination of experimental and informatic approaches, how high order structures form within a cell and we want to identify the characteristics of the interaction graph that promote the formation of a structured cell.

In this first report we presented an initial basic version of a cell automaton that is capable of modeling the dynamics of protein interactions in three dimensions. Our model is presently a naïve one. The cell has no membrane boundary and no compartments. Proteins are fed at a density that is compatible with the estimated physiological protein density, move freely in the cytoplasm and upon meeting have a certain probability of forming a complex according to the rules dictated by a high confidence protein interaction network. It is clear that the more the complexes formed in the ProtNet model reflect the distribution of protein complexes in the cell the more reliable and informative will the biological conclusions that one can draw from such a model. This, however, is difficult to assess because we do not have a full picture of a cell.

In the simulations described in this report the interaction rules are extracted from a yeast high confidence network proposed by Han and colleagues [12]. Albeit "high confidence", this network is incomplete and qualitative. Furthermore, it is biased by interactions that derive from the matrix representations of complexes and, as a consequence, contain a number of interactions that are not direct. The recent publication of two new genomic-scale complex purification experiments [1,3] and a large literature survey to collect information about low-throughput experiments [17] gives us confidence that we should soon be able to compile a much more reliable yeast protein interaction graph enriched for high confidence direct binary interactions. Another important limitation of the experiments reported in this manuscript is the assumption that all the proteins present in the network are equally represented in the cell and that they are indefinitely stable. Protein half lives in a living cell range from a few seconds to many days [18], and the protein abundance in yeast can range from fewer than 50 to more than 1 million molecules per cell [19]. However, since genome wide information about protein concentration in the yeast cell is available this can be readily used to improve the biological reliability of the model [19].

It is somewhat reassuring that, despite the simple nature of the ProtNet model, complexes resembling physiologically important structures like ribosomes and proteasomes are consistently observed (Figure 4). Even in its basic format, ProtNet can readily be used to explore the stochastic nature of protein interactions and how it affects the compositions of the complexes that are formed in the cell. The possibility to vary protein concentrations also allows to evaluate the effects of molecular overcrowding [13] on protein diffusion and complex formation. Finally the dynamic characteristics of the model are particularly suited to assess, for instance, the time required for a cell to reach a new equilibrium stage when either the interaction rules are changed because of a protein modification (i.e. phosphorylation) or when some protein concentrations change because of accelerated degradation or increased gene expression.

## Methods

### The model

As paradigm for the simulation of the dynamics of a PPI network we adopted the stochastic lattice gas [20]. More specifically, we implemented two cell models based on a two and a three-dimensional lattice respectively. In the two-dimensional model the elementary lattice cell has hexagonal geometry while in the three-dimensional one the elementary cell is a cube.

### Protein concentration

Each site of the lattice can be occupied by a single protein. The number of proteins for each molecular species is fixed at the beginning of the simulation. Protein monomers are distributed randomly in the lattice cells. Although, in principle, the concentration of the different protein species can be chosen at will, for instance to mimic the real concentration in the cell, in all the simulations reported in this manuscript each kind of the K protein species is represented by n instances *n ~ N/K *where N is the total number of proteins in the grid. If we consider a grid occupancy ratio p (0 * < p <*1) in a lattice of linear dimensions *L *we have* N = pL*^*d *^where *d *is the lattice dimension, (that is *d *= 2 or 3 for the two and three-dimensional lattice respectively).

At this time we do not model protein synthesis and protein degradation.

### Diffusion and rotation

The simulation iterates for a number of time steps by applying a set of rules that defines the dynamics of the monomers, initially distributed in the lattice, and of the complexes that form when proteins bind.

Empty sites of the lattice are considered *inactive*. However, due to the diffusion and rotation of proteins and complexes, the active sites configuration changes at each time step. More specifically, in the 2D model, at each time step, single proteins and multi-protein structures (*i.e*., complexes) can rotate by 60 degrees around their center of mass in a randomly chosen direction. In the 3D model rotations are by 90 degrees around each of the three axes. Rotations are rigid, that is, in multi-protein structures, the whole complex undergoes a rotation and there is no torsion. For multi-protein complexes, the chance of rotation is inversely proportional to their diameter. As a consequence, "long" protein complexes rotate less frequently. This choice aims at reducing the impact of the dramatic changes in the configuration of active sites caused by the rotation of long multi-protein complexes.

The diffusion coefficient of a molecule in a dilute solution is a complex function of its shape: *D = (1/f)kT*, where *f *is the frictional coefficient, *k *the Boltzman constant and *T *the absolute temperature. For a spherical object the frictional coefficient increases linearly with the inverse of the radius. In the current implementation of our model we do not input information about protein masses and shapes. As a consequence all proteins are assumed to have identical diffusion coefficients. In our simulations, monomers have the highest probability of moving to one of the neighboring elementary cells at each time step. Multimers diffuse more slowly (see below).

Having set the linear dimension of the lattice cell to 5 nm, the diameter of an average globular protein, we can estimate the time step of the simulation.

In the limit of large t, the mean-square displacement <x^2^> of a lattice gas is <*x*^2^> = *2dD(p)t*

where *d *is the dimension of the lattice (2 or 3 in our case) and *D *is the diffusion coefficient that is a function of the concentration *p*. If *l *is the size of the random walk step (5 nm in our model), *M *is the number of steps and *t*_0 _is the elementary time step (*M = t/t*_*0*_) we have

*2dD(p)t = l*^2^*t/t*_*0*_

that is, for a 2D lattice:

*t*_*o *_= *l*^2^*/4D(p)*

Assuming an average diffusion coefficient of 10^-7 ^cm^2^sec^-1^, this results in a time step of 6 × 10^-7 ^s.

Multimeric complexes have larger frictional coefficients and therefore move more slowly. However, in the calculation of frictional coefficients, we do not take into account the shape of the complexes but we assume an inverse linear relation between the diffusion coefficients and the complex sizes, that is the number of monomers forming a complex.

When a monomer diffuses and when a multi-protein complex diffuses or rotates, another single or multi-protein complex can be "hit" if it occupies a "target" site. In this case, the moving protein (or complex) pushes the still protein (or complex) with a probability that is a function of the ratio between the masses of the hitting and hit protein/complex. In particular, the probability that the hitting complex with mass m_1 _pushes the still complex with mass m_2 _is

p = e[-(m2/m1)*log2]

as a consequence, if m_1 _>> m_2_, on average the hitting complex pushes m_2 _whereas, if m_1 _<< m_2 _and m_1 _hits m_2_, then m_1 _stops. In the case m_1 _~= m_2_, the probability the push succeeds is 1/2. Note that this mechanism is applied recursively in situations where a colliding protein or complex m_1 _pushes m_2 _which in turn pushes m_3 _and so on.

### Association and dissociation

At each time step new complexes can form and existing complexes can break down into smaller complexes according to experimental interaction rules provided as input to the model. Two proteins residing in neighboring lattice sites may interact and form a new complex depending on whether they are linked by an edge in the input interaction graph.

Monomers have six *binding *sites corresponding to the six sides of the hexagon in the 2D model or to the six cube faces in the 3D version of the model. Therefore, during a simulation, a single protein can bind to, at most, six other partners. Proteins do not compete for binding to the same partner and association is only limited by the number of edges, 6 in both 2D and 3D models. However, to prevent the formation of very large complexes containing multiple copies of the same protein, we implemented the *ad hoc *rule that a complex may contain only one copy of any protein species. Binding is stochastic and the probability of forming a complex is a function of an association probability that is related to the association rate constant (k_on_) of the interacting protein pair. After association, a complex moves as a single entity. Since for most protein pairs the value of the association/dissociation constants is unknown, we set it equal to an *average *value that is the same for all proteins.

**Figure 6:**
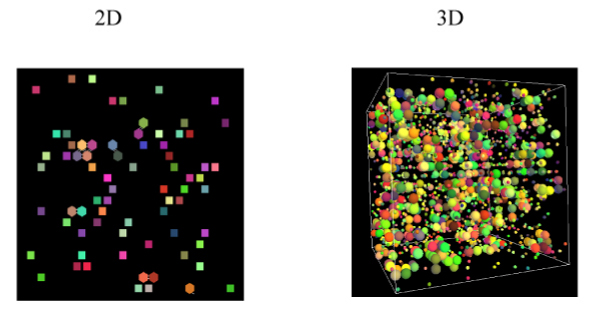
**Graphic representation of the 2D and 3D models**. The two cartoons are visual representation of the 2D and 3D ProtNet models. The different colors of the elements in the two lattices illustrate different protein species. Although all the sites in the 2D model have a hexagonal geometry, proteins that are not yet involved in interactions are depicted as squares. Similarly, in the 3D model, free monomeric proteins are depicted as smaller spheres.

The model has been implemented by using the C language for performance reasons and for maintaining full control of memory management that can be an issue for simulations of large lattices. The resulting code is portable and runs under any major operating system (Linux, Windows, MacOS X, *etc*.). A simulation step, with a lattice containing approximately 10^6 ^sites at a protein concentration of 20%, takes approximately 0.25 seconds on an Intel(R) Pentium(R) 4 Mobile CPU 1.60 Ghz. (Along with the simulator, a visualizer has been implemented for both the 2D and the 3D version of the model (Figure 6). The visualizer is based on the OpenGL Application Programming Interface. By means of the visualizer, it is possible to look at the dynamic processes leading to the formation of protein complexes in *real time *during the simulation. The simulator comes with a portable Graphics User Interface (GUI) that simplifies the definition of input data and simulations management.)

## Authors' contributions

MB has coordinated the development of the software and has developed the graphic interface.  FC has contributed to the ProtNet software and performed several simulations.  AF has written the ProtNet software.  CG has performed the analysis of several simulations.  MT has contributed the yeast network.  GC has conceived the project, coordinated it and written the manuscript.

## Supplementary Material

Additional File 1 High confidence yeast interaction network. Click here for file
